# Identification of frequent MTNR1B methylation in breast cancer following the application of high-throughput methylome analysis

**DOI:** 10.1186/1471-2164-15-S2-P44

**Published:** 2014-04-02

**Authors:** Shylu Mathew, Adnan Merdad, Jaudah Al-Maghrabi, Ashraf Dallol

**Affiliations:** 1 Center of Excellence in Genomic Medicine Research, King Abdulaziz University, Jeddah, KSA; 2 Department of Surgery, King Abdulaziz University Hospital, King Abdulaziz University, Jeddah, KSA; 3 Department of Pathology, King Abdulaziz University Hospital, King Abdulaziz University, Jeddah, KSA

## Background

Breast cancer is the main cancer type affecting women in the Kingdom of Saudi Arabia. The relatively young age of onset in this population could be explained by the interplay between common genetic susceptibility background substantiated by increased consanguinity and epigenetic aberrations caused by the shift in life style experienced in this region [1]. Genomic screening of breast cancer patients is beneficial in identifying underlying variants that could explain increased susceptibility to breast cancer. However, it is important to understand the epigenetic aberrations associated with breast cancer in order to shed light on its etiology and identify possible treatments. To this end, we have performed MBD-Seq on a cohort of breast cancer samples that led to the identification of tumor-specific methylation of the MTNR1B promoter in a significant number of breast cancer cases from Saudi Arabia.

## Materials and methods

Methyl binding domain-sequencing (MBD-Seq) was applied on DNA extracted from surgically-resected breast tumors using the MethylMiner™ kit from Invitrogen followed by fragment identification using next generation sequencing on the SOLiD platform (Applied Biosystems). Determination of methylation frequency and correlation with clinicopathological parameters was performed using the MethyLight assay on DNA extracted from FFPE tissues. Fisher’s exact test and univariant Kaplan-Meier survival analysis were applied where p<0.05 considered statistically significant.

## Results

MTNR1B methylation frequency in breast cancer is 35% (n=157). MTNR1B methylation was largely limited to the IDC, ILC and DCIS subtypes. Furthermore, MTNR1B methylation is significantly associated with histological grade I of breast cancer (p=0.019, n=128). The association of MTNR1B methylation and disease-free or specific survival is investigated.

## Conclusions

Finding significant levels of methylation of a key circadian clock gene as the MTNR1B in a tumor-specific fashion may provide an intriguing evidence to the role of environmental factors (day-night cycles) and breast cancer development.

This project is funded by KACST grant ARP-29-292.
